# Modulation of MICAL Monooxygenase Activity by its Calponin Homology Domain: Structural and Mechanistic Insights

**DOI:** 10.1038/srep22176

**Published:** 2016-03-03

**Authors:** Saif S. Alqassim, Mauricio Urquiza, Eitan Borgnia, Marc Nagib, L. Mario Amzel, Mario A. Bianchet

**Affiliations:** 1 Department of Neurology and Department of Biophysics & Biophysical Chemistry, Johns Hopkins University School of Medicine, 725 North Wolfe St., Baltimore, MD 21205, USA; 2 Structural Enzymology and Thermodynamics Group, Department of Biophysics & Biophysical Chemistry, Johns Hopkins University School of Medicine, 725 North Wolfe St., Baltimore, MD 21205, USA

## Abstract

MICALs (Molecule Interacting with CasL) are conserved multidomain enzymes essential for cytoskeletal reorganization in nerve development, endocytosis, and apoptosis. In these enzymes, a type-2 calponin homology (CH) domain always follows an N-terminal monooxygenase (MO) domain. Although the CH domain is required for MICAL-1 cellular localization and actin-associated function, its contribution to the modulation of MICAL activity towards actin remains unclear. Here, we present the structure of a fragment of MICAL-1 containing the MO and the CH domains—determined by X-ray crystallography and small angle scattering—as well as kinetics experiments designed to probe the contribution of the CH domain to the actin-modification activity. Our results suggest that the CH domain, which is loosely connected to the MO domain by a flexible linker and is far away from the catalytic site, couples F-actin to the enhancement of redox activity of MICAL_MO-CH_ by a cooperative mechanism involving a *trans* interaction between adjacently bound molecules. Binding cooperativity is also observed in other proteins regulating actin assembly/disassembly dynamics, such as ADF/Cofilins.

Growing axons are guided to their appropriate targets by extracellular attractive or repulsive cues that are essential for proper neuronal growth and development, rewiring, fasciculation/defasciculation, and nerve regeneration after injury^1^,^2^. Semaphorins, the most well characterized class of external repulsive guidance molecules, interact with Plexin and neuropilin receptors on axonal growth cones^3^. Upon Plexin interaction with extracellular semaphorins, its cytosolic domain recruits and activates MICAL (Molecule Interacting with CasL); this activation promotes reorganization of the cytoskeleton and subsequent growth cone collapse^4^,^5^. Since its initial identification in T-cells^6^, MICAL has also been found in a variety of neuronal and non-neuronal cell types in which it controls cytoskeletal dynamics^7^,^8^.

Three MICAL isoforms (MICAL-1, -2, and -3) have been identified in vertebrates^4^,^5^. They have high overall sequence identity (1–2: 56%, 1–3: 56%, and the highest for 2–3: 65% in mouse MICALs). MICALs are large cytosolic proteins with an N-terminal flavoprotein monooxygenase (MO) domain containing an FAD cofactor followed by a variable number of protein-interaction domains^3^. MICAL-1 combines the catalytic MO domain (residues 1–484) with three other domains thought to be important for modulating MICAL’s activity and/or interaction with substrates: 1) a CH domain (residues 511–615), 2) a Lin-11 Isl-1 Mec-3 (LIM) domain (residues 666–761)^9^, and 3) a C-terminal region containing a coiled-coil Ezrin Radixin Moesin (ERM) domain^10^,^11^. In addition, MICAL-1 contains a poly-proline PPKPP sequence (residues 830–834) that binds the SH3 domain of CasL^6^.

In mouse MICAL-1 (mMICAL-1, M_W_: 117 kDa, 1048 amino acids), the MO domain (MICAL_MO_) has been shown to reduce molecular oxygen to H_2_O_2_, with a ~70-fold preference for NADPH over NADH as the source of reducing equivalents^12^. The structure of mMICAL-1 MO domain, determined by x-ray diffraction^12^,^13^, contains many of the features common to FAD-containing monooxygenases such as ρ-hydroxybenzoate hydroxylase (pHBH) with one major difference: the cavity that connects to the active site is larger in MICAL_MO_ than in pHBH and could potentially accommodate a protein substrate; in contrast, pHBH substrates are small molecules that can be easily accommodated in a small cavity. In the structure determined in the presence of NADPH, the isoalloxazine ring of the reduced FAD adopts an “in” conformation, in which it is less accessible to water and to O_2_^13^. The CH and other domains of MICAL could play a role in keeping the active site less accessible to solvent, resembling pHBH and monoamine oxidases^14^.

CH domains have a highly conserved architecture comprised mainly of α-helices^15^. They carry out diverse functions in cytoskeleton binding and signalling^16^. The α-actinin and spectrin protein families, both of which are known to cross-link actin filaments, interact with actin filaments via two tandem CH domains (classified as type-1 and -2). Some proteins, such as calponin and IQGAP, contain single CH domains classified as type-3. Smoothelins and RP/EBs^5^ as well as MICALs contain a single type-2 CH domain. However, MICALs are unique in the sense that their CH domain is adjacent to a domain containing catalytic activity.

Hung *et al*. reported that expression of *Drosophila* MICAL constructs containing either the MO alone or MO plus the CH domain result in proteins with a redox activity that alters actin polymerization dynamics; specifically, they oxidize Met 44 of F-actin, leading to destabilization and disruption of actin filaments^17^,^18^. However, although the MICAL_MO_ alone is sufficient to bind and oxidize F-actin *in vitro*, *in vivo* full-length *Drosophila* MICAL (dMICAL) with only the CH domain deleted (MICAL^∆CH^) shows defects in actin processing and motor axon guidance^17^,^18^. For example, in *Drosophila* bristles (an *in vivo* single cell model of MICAL-mediated F-actin depolymerization), dMICAL co-localizes with F-actin, and the CH domain participates in this localization, as suggested by the observation that MICAL^∆CH^ and MICAL^ΔPIR^ —lacking the Plexin interacting region—are unable to co-localize with F-actin^17^. *Drosophila* MICAL^∆CH^ mutants also show dominant defects in axon defasciculation and guidance^18^,^19^. Furthermore, the CH domain is required for MICAL-mediated cytoskeleton organization *in vivo*^20^,^21^. Interestingly, the CH domain of MICAL (MICAL_CH_) participates in these actin–associated functions even though the isolated domain does not bind F-actin *in vitro*^20^,^21^. Although it is clear that the MICAL_CH_ is functionally important, its relation to the MO domain and how it modifies the MICAL_MO_ activity toward F-actin remain obscure.

The structures of the isolated MICAL_MO_^12^,^13^ and MICAL_CH_^20^,^21^ have been determined, but they do not provide information about how the CH domain may contribute to connect MICAL activity to interactions with the cytoskeleton. Understanding the modulation of the MICAL_MO_ catalytic activities by the CH domain requires the determination of the structure of a MICAL fragment containing the entire MO and CH domains. Here, we report the structure of a protein containing the MO and CH domains of mMICAL-1 (MICAL_MO-CH_; residues 2 to 615) determined using x-ray diffraction and small-angle x-ray scattering (SAXS). The structure provides the basis for a model for the oxidation of Met 44 of actin by MICAL. In addition, using steady-state kinetics, we demonstrate that the CH domain of mMICAL-1 enhances both recognition of F-actin and NADPH as well as the NADPH oxidase of MO activity in the presence of F-actin. The high level of conservation of the MICAL MO and CH domains in species ranging from *Drosophila* to *Homo sapiens* suggest that structural and functional inferences made here are likely to be applicable to other MICALs, including MICAL-2 and -3.

## Results

### Structure Determination

**Figure 1:**
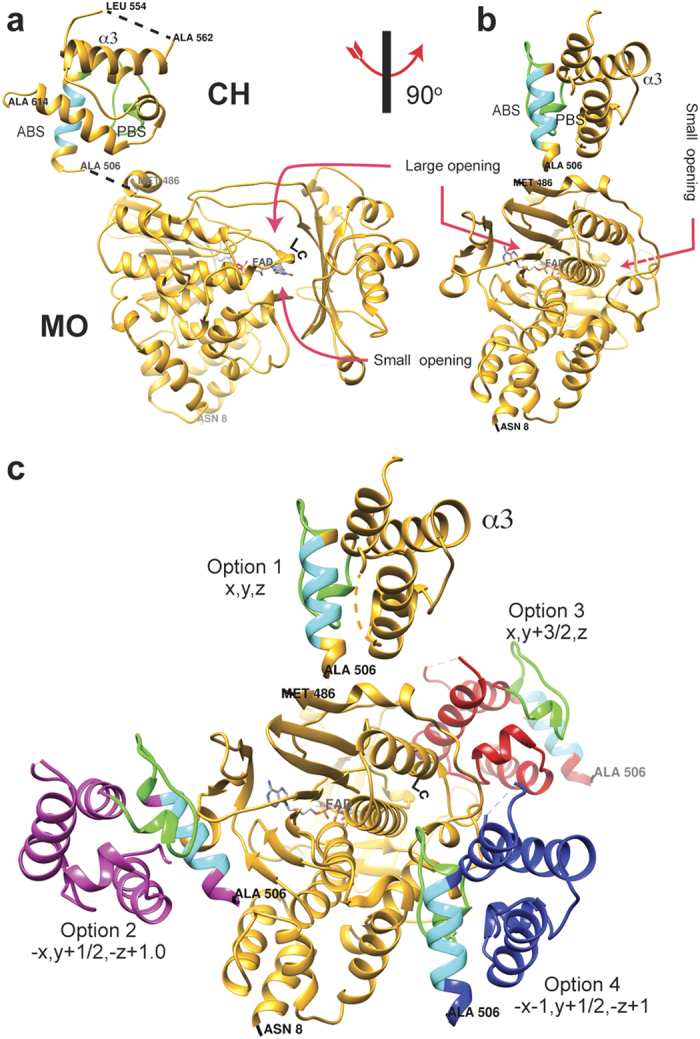
Structure of MICAL_MO-CH_ and its different asymmetric unit choices. (**a)** Structure of MICAL_MO-CH_ (option 1). Residues of the actin binding sequence (ABS; 511-EELLHWCQE-519) of the CH domain are shown in cyan, and residues of the PIP_2_ binding segment (521-AGFPGVHVTDFSSSWAD-538) are in green. (**b)** Another view of the structure rotated 90^o^ around the vertical axis. (**c)** A view showing the four possible choices of asymmetric unit with the symmetry operations that relates to option 1. MO and CH domains of option 1 are colour in yellow. CH domains of the other options are coloured in: blue (option 2), red (option 3), magenta (option 4). The option number is indicated in the figure. FAD cofactor carbon atoms are shown as green sticks.

**Figure 2:**
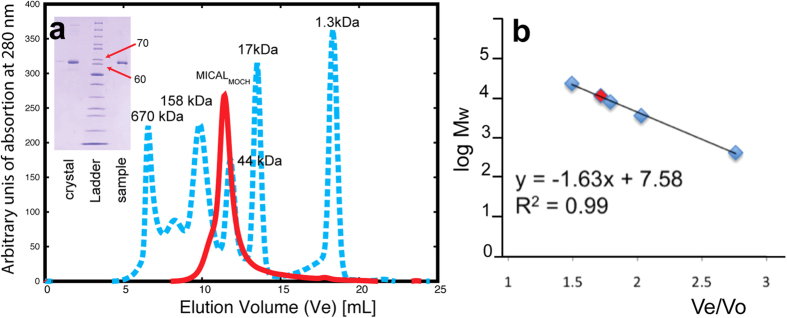
MICAL_MO-CH_ is an intact molecule in solution and in the crystal. (**a**) Molecular weight characterized by size exclusion chromatography MICAL_MO-CH_ eluted as a monomer of 61.4 kDa (red line) in a size exclusion chromatography using a Superose-12 10/300GL (GE HealthCare) column. Standards are shown in blue, with the molecular weights (Mw) in kDa indicated. (**b**) Logarithmic plot of the Mw for the standards the ratio between elution volume (Ve) and column exclusion volume (Vo). The pink diamond mark indicates the MICAL_MO-CH_ elution volume observed. (Inset) SDS-PAGE analysis of MICAL_MO-CH_ crystals. Lane 1: crystals dissolved in SDS running buffer after washing with crystallization mother liquor; lane 2: 10, 15, 20, 25, 30, 40, 50, 60, 70, 80, 90, 100, 110 kDa molecular weight ladder and lane 3: MICAL_MO-CH_ after purification.

The structures of two crystal forms, native 1 and 2 (Table 1), show electron density for residues 8–486 of the MO domain and residues 506–554 and 562–614 of the CH domain (Fig. 1). Although no electron density was observed for the 19-residue linker connecting these domains, SDS-PAGE analysis of re-dissolved crystals shows that no cleavage had occurred during purification or crystallization that could explain the missing linker (Fig. 2a inset). Although the native 1 crystal was made in the presence of a large amount (19 mM) of a synthetic actin “D-loop” peptide (residues 39 to 52 of actin), it did not show additional electron density for this ligand (hence was named “crystal 1”). The native 1 structure was refined to an R_work_ and R_free_ of 0.25 and 0.30 using data to 2.31 Å resolution (Fig. 1). For the native 2 crystal, obtained without the addition of actin derived peptide, the R_work_ and R_free_ were 0.23 and 0.28 for data to a resolution of 2.9 Å (Table 1). Size exclusion chromatography showed that, under the conditions used for crystallization MICAL_MO-CH_ is a monomer in solution (Fig. 2). In agreement, both crystal forms analysed contain one single chain monomer per asymmetric unit.

### Structure of the MO domain

MICAL_MO_ contains a Rossman β-α-β fold with a GXGXXG motif, a sequence common in NADPH-dependent FAD-containing oxidoreductase enzymes. In both crystal forms the FAD cofactor is oxidized and accessible through two openings connected to the active site, one large and one small (Fig. 1a). Alignment of the MO domain of the MICAL_MO_ crystal structure (PDB 2BRA^12^) to the same domain of the MICAL_MO-CH_ structure shows a 0.5 Å RMSD for 422 Cα atoms. Only small localized differences are observed (Supplementary Fig. S1).

### Structure of the CH domain

**Figure 3:**
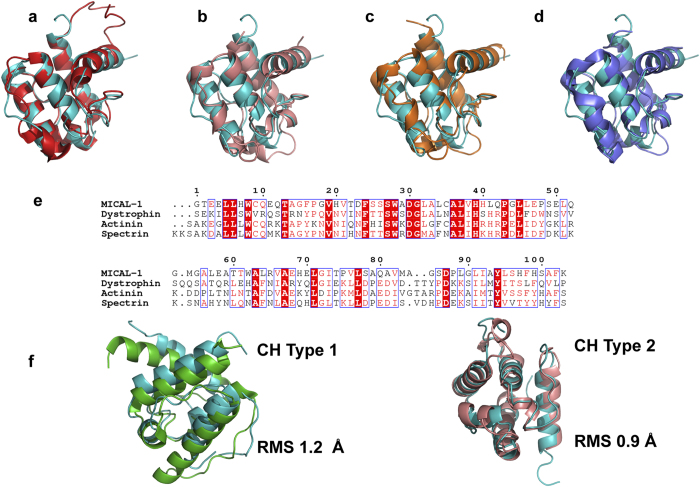
Structure and sequence alignment of MICAL_CH_ with characterized CH domains. The MICAL_CH_ (cyan) align with known type-2 CH domains from (**a)** human MICAL_CH_ (1WYL, coloured red), (**b)** α-actinin (2EYI, coloured pink), (**c)** spectrin (1BKR, coloured orange), and (**d)** dystrophin (1DXX, coloured blue). All four CH type 2 domain align with an RMSD of ~1 Å with most if not all Cα atoms. (**e)** Sequence alignment of MICAL_CH_ with the same proteins in (**b**–**d**) panels. (**f)** Structural alignment of MICAL_CH_ with the CH type 1 (rigth) and type 2 (left) of actinin (PDB 2EYI); MICAL_CH_ coloured in cyan, actinin CH type-1 in green, and actinin CH type-2 in pink.

**Figure 4:**
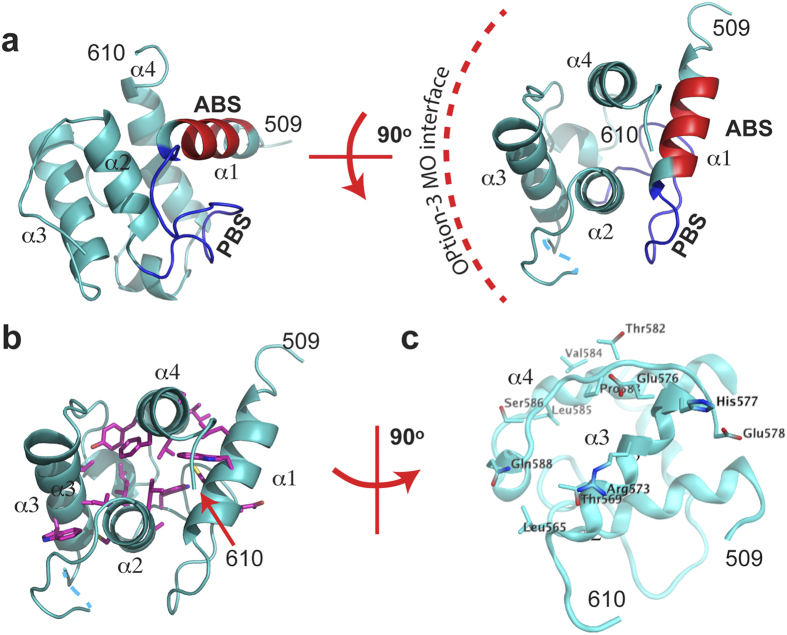
Overview of the CH domain from MICAL_MO-CH_. (**a)** top and side views of the CH domain. Residues comprising the conserved ABS found in type-2 CH domains are shown in red. Residues that match the PIP_2_ binding site in type-2 CH domains are in blue. (**b)** CH domain (same view as panel **b**), highlighting the residues that makes up the hydrophobic core – represented as magenta sticks.

The structure of the CH domain of MICAL_MO-CH_ is highly similar to that of the isolated CH domain of human MICAL-1 (PDB 1WYL and 2DK9) determined by NMR methods^20^ (RMSD of 0.8 Å for 82 Cα atoms; Fig. 3a). As in other CH domains of the same type found in actin-binding proteins (Fig. 3a–d), the CH domain of MICAL_MO-CH_ consists of three α-helices packed as a parallel bundle, with a fourth α-helix that is perpendicular to the other three (Fig. 4). As expected from its sequence, the structure of MICAL_CH_ aligns better to the type-2 CH domain of actinin (PDB 2EYI) than to the type-1 (Fig. 3). Key hydrophobic residues important for helix packing and for binding to F-actin are conserved (Figs 3d and 4b)^15^,^20^,^21^.

The CH domain of MICAL_MO-CH_ also shows the other conserved features found in type-2 CH domains of actin-binding proteins: a conserved actin-binding segment (ABS; Fig. 4) found in the helix perpendicular to the other three, and a conserved PIP_2_ binding site (PBS; Fig. 4)^15^. In the MICAL_MO-CH_ structure, these segments are exposed to solvent and are free to interact with other proteins or the membrane (Fig. 1c).

### Connectivity between MO and CH domains

**Figure 5:**
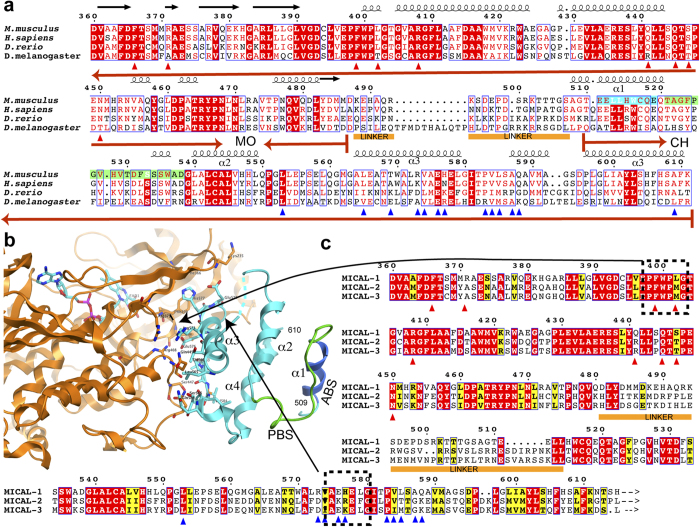
Sequence alignments of MICAL-1 CH domain homologues. MICAL-1 sequences from the indicated organisms were aligned from near the C-terminus of the MO (aa 458) to the end of the CH domain (aa 615). Residues in red boxes are conserved among species. The residues participating in the interaction between the CH and the MO domains of the option 3 are marked with a triangle, coloured red and blue in the MO domain and CH domain, respectively. Actin binding sequence (ABS) and PIP binding region (PBS) are coloured cyan and green respectively only in the mouse sequence. The missing linker is indicated by a yellow bar bellow the sequence. The secondary structure elements observed in the structure reported here are displayed on top of the sequences aligned. Alignment was performed using ClustalW^39^. Figure prepared with ESPript^40^.

There is no observable electron density in either of the two crystal forms for the 19-residue linker that connects the MO and CH domains. The sequence of this linker region (residues 488–506) is not well conserved in the different MICAL-1s (Fig. 5a), in stark contrast to the MO (residues 7–487) and the CH domain (residues 507–611) of MICAL-1, which show highly conserved sequences from *Drosophila* to *Homo sapiens* (mouse sequences are 91% identical and 93% similar to the MICAL_MO-CH_ region of human MICAL-1; Supplementary Fig. S2). Based on sequence, the linker region is predicted to be unstructured.

**Figure 6:**
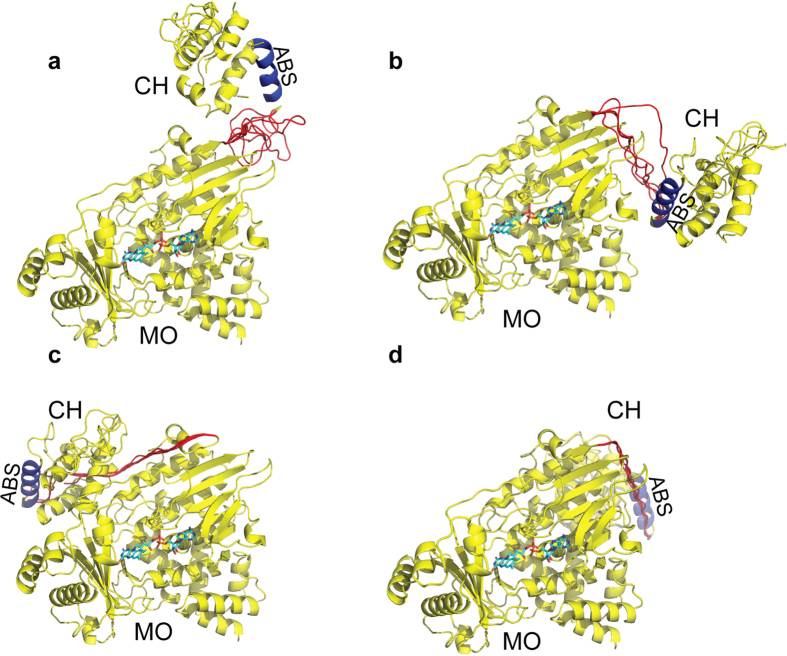
Asymmetric unit options of crystal 1 showing the modelled linkers between MO and CH domains. (**a**–**d)** are option 1 to 4 respectively. The five *ab-initio* traces of the linker generated by the program Modeller^41^ are coloured in red and the ABS of the CH domain in blue. The FAD carbon atoms are coloured in cyan.

Lack of density for the peptide connecting between the two domains introduces an uncertainty as to which of the CH domains among symmetry mates belongs in the same molecule as a given MO. Examination of the asymmetric unit reveals that, given the length of the linker (19-residues), the MO could be connected to any one of four possible CH domains (options 1 to 4, shown in Figs 1c and 6). Equivalent options are present in both crystal forms.

Alignment of the structures of the MO domains of native 1 and native 2 shows that in the two crystal forms the positions of the four CH domains from alternative asymmetric units are shifted relative to the MO domain. Aligning the MO domains of the different options, the root mean square distance between the 103 Cα atoms of the four CH domain options are the largest for option 1 and 4 (8.63 and 8.0 Å, respectively), while those for options 2 and 3 are significantly lower (4.5 Å and 4.6 Å, respectively). In both crystal forms, options 1, 2, and 4 place the CH domain far away from the catalytic site (Fig. 6a,b,d), making minimal contact with the MO domain as indicated by the small buried surface area (Table 2). The CH domain of option 3 in both forms buries by far the largest surface area of all four options (1015 Å^2^ and 901 Å^2^; for native 1 and 2 respectively, Table 2). In this option, the CH docks on the side of the small entrance to the catalytic site without obstructing it, but it contacts the catalytic site loop Lc (Fig. 1a). Lc residues, in particular Trp 405, form the cavity that the isoallozaxine rings of the (hydroxy)peroxi-C4a-FAD occupies when it swings-in (Figs 1a and 5b)^12^,^13^. This entrance may channel the substrate to the C4a-(hydro)peroxy- intermediate site when the FAD is reduced (Fig. 1a,b). In option 3, the interface on the CH domain interacting with the MO domain is formed by CH domain helix α3 and the loop connecting this helix with α4 (Fig. 4). This interface involves Leu 553, Leu 565, Thr 569, Arg 573, Val 574, Glu 576, His 577, Glu 578 of α3, and Gly 580, Thr 582, Pro 583, Val 584, Ser 586, and Gln 588 of the loop (Fig. 4c). The MO side of the interface involves Lys 235, Arg 371, Phe 399, Leu 402, Arg 408, Gln 441, Leu 442, Ser 444, Gln 445, Ser 447, and Asn 450 (Fig. 5b). Residues on the MO domain participating in the MO-CH interface are well conserved in vertebrates —to a lesser degree in *Drosophila—* (Fig. 5a) as well as among mouse MICAL isoforms (1–2: 67%,1–3: 62.5%, and notably 2–3: 96% of sequence identity). Residues of the CH domain that participate in the interface show lower sequence identities between MICAL-2 or -3 and MICAL-1 (Fig. 5c; 1, 2 and 1, 2, 3: 28.6%), but are highly homologous to each other (Fig. 5c; 2, 3: 77%). The CH domain residues that participate in this interface, although they have lower sequence identity with each other than their counterparts in the MO domain, they do conserve hydropathy and size characteristics across species and isoforms (Fig. 5a,c).This interaction is mostly polar in nature, and shows significant charge complementarity. Residues Arg 408 and Glu 576, which form a salt bridge, are conserved from *Drosophila* to humans (Fig. 5a) and among isoforms (Fig. 5c). Also, the NH and the amine nitrogen of Lys 235 form a hydrogen-bonding network with the main chain carbonyl and the side chain of Glu 578. Another hydrogen bond is observed between the side chain of Ser 447 and the carbonyl oxygen of Pro 583.

To examine whether it was possible to connect the MO and CH domains of MICAL_MO-CH_ in all four options of native 1, five independent models of the 19-amino acids linker were generated for each option using the program MODELLER^22^. The models show that it is possible to connect the two domains with a 19-amino-acids linker only for options 1, 2 and 4 (Fig. 6). Linkers for option 3 in mouse MICAL_MO-CH_ were predicted to be in highly strained implausible conformations (Fig. 6c). However, the linkers of Drosophila MICAL, and mouse MICAL-2 and -3 are longer than that of MICAL-1 (11, 4 and 6 residues, respectively; Fig. 5c), which could allow these enzymes to reach an intermolecular association equivalent to option-3.

### Small angle X-ray scattering (SAXS) of MICAL_MO-CH_

**Figure 7:**
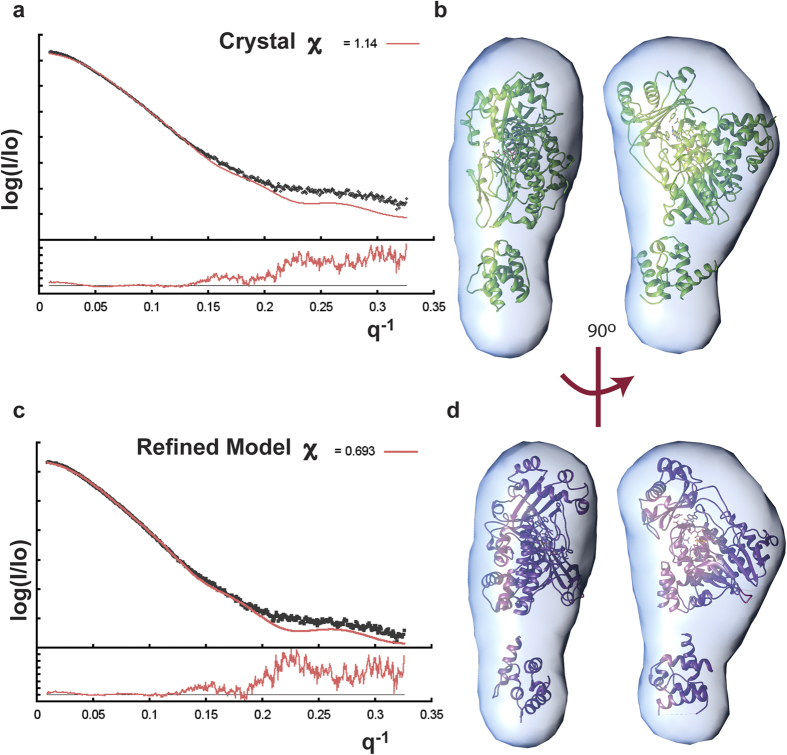
Agreement between models from x-ray scattering and diffraction data. (**a**) Adjustment of the experimental SAXS profile of MICAL_MO-CH_ (black scattered points) by the calculated profile (red solid line) of the crystal structure option 1 (green solid line). (**b)** Top and side view of the fitting of the *ab-initio* envelope by the crystal structure of MICAL_MO-CH_ option 1. (**c)** Adjustment of the same experimental profile (scattered points) by the crystal structure model refined by Sasref (ATSAS) against the SAXS data. **(d)** Top and side view of Sasref-refined model fitting of the *ab-initio* envelop. The experimental scattering profile was obtained using the average scattering of three different exposures (0.5, 1, and 2 sec) of a solution of MICAL_MO-CH_ at 7 mg/ml. All the theoretical profiles were generated using FoXS^42^,^43^. *Ab-initio* envelope fitting was performed using Supcomb (ATSAS) with “native 1” structure enabling the enantiomorphism option.

SAXS data were obtained to provide independent information about the domain arrangement and to help resolve the uncertainty in the connectivity. The radius of gyration (R_g_) of MICAL_MO-CH_ estimated from the SAXS data, is 31 Å and the D_max_ is 122 Å (Fig. 7 and Supplementary Fig. S3). The SAXS estimated particle mass, 70.1 kDa, corresponds to the mass predicted from the sequence of the MICAL_MO-CH_ monomer (68.5 kDa,) within 4.4% of error (Table 3). The experimental R_g_ is significantly larger than that estimated for option 3 (R_g_ = 24.5 Å; Table 3). The other three options predict an R_g_ significantly larger than that of option 3, but, still shorter than the experimental value (Table 3). Option 1 has the smallest discrepancy between predicted and experimental scattering data (χ^2^ = 1.5; R_g_ = 28.5 Å) and a close fit to the low-resolution *ab-initio* SAXS model (Table 3, Fig. 7a). The low-resolution *ab initio* model (envelope) and rigid body refinement of option 1 using SAXS data indicate a slightly greater separation between domains in solution than in the crystal (Fig. 7c,d).

### Activity of MICAL_MO_ and MICAL_MO-CH_ in the presence of F-actin

**Figure 8:**
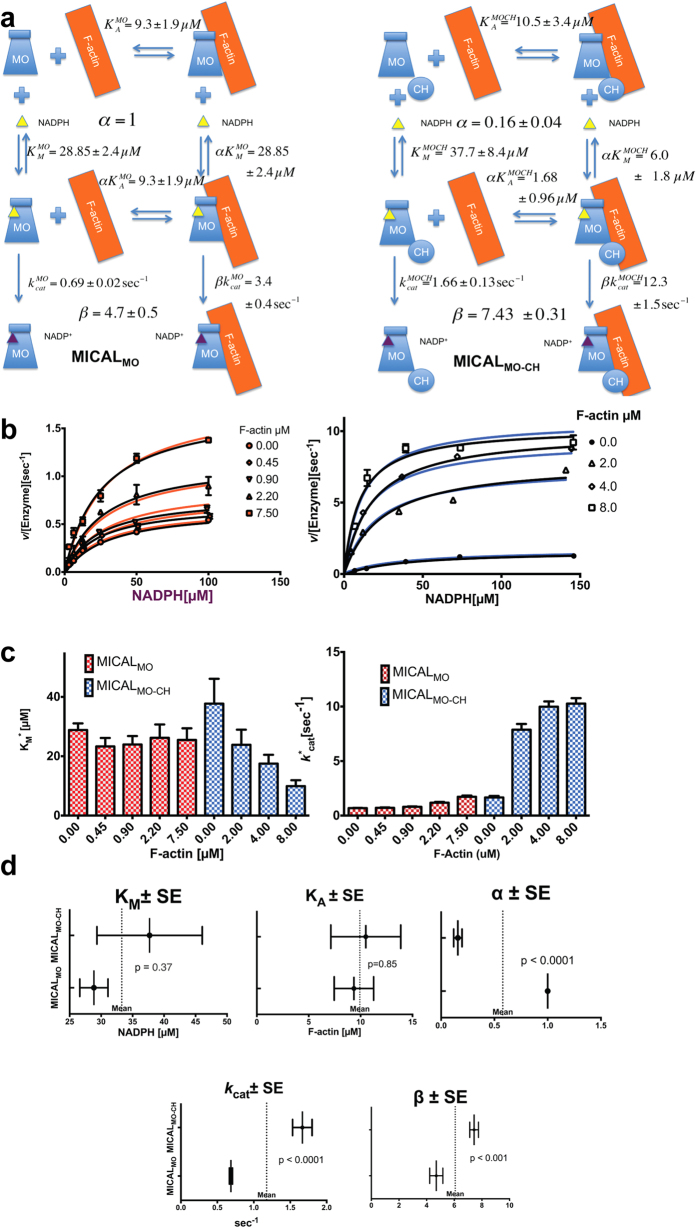
Steady state enzyme kinetics analysis. (**a)** Rapid equilibrium diagrams for the system enzyme (MICAL_X_)/substrate (NADPH)/activator (F-actin). (Left) for MICAL_MO_ and (right) for MICAL_MO-CH_. The equilibrium constants and rates in each branch are shown with their SE calculated from the non-linear fit of Equation. [1] to the initial rate of NADPH oxidation by MICAL_MO_ and MICAL_MO-CH_ in presence of F-actin. (**b)** Steady state velocities and their fitting with Equation (1) (color traces) and Equation (2) (black traces). The symbol error-bars represent the standard error of the mean SEMs. (**c)** Apparent 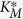 and 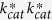 of both constructs obtained by the fitting the initial velocities for each value of [F-actin] with equation (2). (**d**) Parameters of the global fitting of MICAL_MO_ and MICAL_MO-CH_ using the non-essential activator (F-actin) Equation (1), the parameter α was constrained to 1 in the MICAL_MO_ dataset. Error bars represent standard errors (SEs). All fitting and statistical tests were performed using the program Prism 6 (GraphPad Inc.).

F-actin behaves as a non-essential activator of NADPH-oxidation by MICAL_MO_ and MICAL_MO-CH_: the addition of F-actin increases the rate of reaction but catalysis still takes place in its absence, albeit at reduced rate (Fig. 8). Consequently, the initial velocities of the NADPH-oxidation (***v***) follow the typical non-essential activator steady-state kinetics scheme shown in Fig. 8a. These velocities are well represented by Equation (1), a hyperbolic function of the substrate concentration and activator ([*NADPH*] and [*F-actin*], respectively) at given concentrations of enzyme ([*Enzyme*]). In Equation (1), the coupling parameter α measures the synergy between substrate affinity and binding of the activator or *vice versa* (α = 1 no coupling, α < 1 positive coupling, etc.), and β measures the acceleration factor of the turnover-number when the ternary complex (enzyme-substrate-activator) is formed. The apparent Michaelis-Menten constant (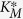) of the substrate electron donor and apparent turnover-number of NADPH oxidation (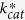) were also obtained by fitting these velocities at a given [F-actin] with the simple Michaelis-Menten enzyme/substrate steady-state kinetic equation (2) (Fig. 8b, Table 4). For MICAL_MO_ the 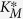 values vary only slightly and not regularly, indicating that actin does not affect the NADPH binding to the isolated MO domain (Table 4). Therefore, the observed kinetics for MICAL_MO_ can be fit with a constant 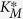 (28.8 ± 2.4 μM) across the range of [F-actin] analysed (Fig. 8c, Table 4). The observed independence of the binding constants (Ks) with the concentration of the other substrate is modeled with a value of α equal to 1 in Equation (1). In contrast, for MICAL_MO-CH_ the 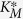 values decreases when [F-actin] increases (Fig. 8c and Table 4), suggesting a value of α different from 1 in Equation (1). Data were fitted to Eq. (1) restraining α to 1 in the case of MICAL_MO_ and allowing α to vary in the case of MICAL_MO-CH_. Both sets show good fit to the experimental values (Fig. 8b). The *K* constants fitted for MICAL_MO_ and MICAL_MO-CH_ are not significantly different (Fig. 8d; K_M_ of 9.3 ± 1.9 μM *vs* 10.5 ± 3.4 and K_A_ of 28.8 ± 2.4 μM *vs*. 37.7 ± 8.4, respectively). In contrast, for MICAL_MO-CH_ the data are fitted with a value α of 0.16 ± 0.04, indicating strong cooperativity between binding NADPH and actin.

In both cases, 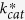 increases with the F-actin (activator) concentration, although the magnitude of the change is significantly greater for MICAL_MO-CH_ than that for the isolated MO domain (Fig. 8c,d). The rate of NADPH oxidation by either protein increases in the presence of actin, but the increase is smaller for MICAL_MO_ (β = 4.7 ± 0.5 for MICAL_MO_; β = 7.43 ± 0.31 for MICAL_MO-CH;_ Fig. 8d). The dependence of the *K*s for NADPH and actin on each other’s concentration in MICAL_MO-CH_ is the major difference between the two proteins (α = 0.16 for MICAL_MO-CH_ vs. 1.0 for MICAL_MO_).

### Model of MICAL_MO-CH_/F-actin interaction

As the D-loop of actin is part of a large oligomeric filament (F-actin) it raises the question of whether it is accessible to the active site of MICAL. For a *direct oxidation* to be possible, the sulfur atom of Met 44 (well below the surface in F-actin) should be positioned within ~3 Å of the C4a (hydroxyl)peroxy-FAD- intermediate so that oxidation can occur. Thus, a conformation should be found in which the actin D-loop of a filament comes close to the C4a of the FAD cofactor in a way that allows the oxidation to take place. This conformation should be accessible from observed structures without breaking any covalent bonds or affecting the structural integrity of either actin or MICAL.

**Figure 9:**
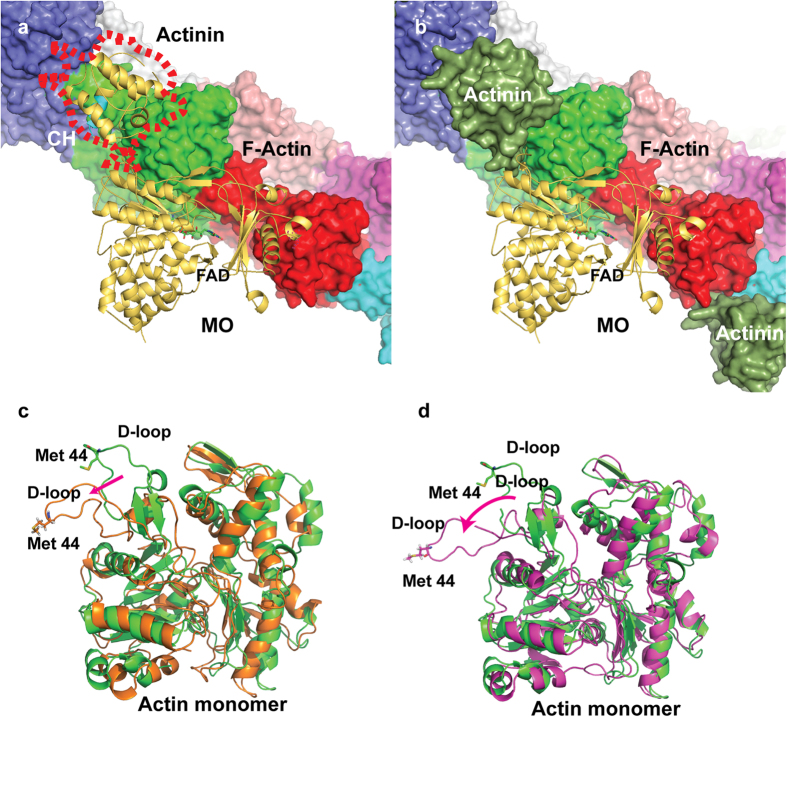
Model of MICAL_MO-CH_/F-actin interactions. (**a**) Starting model used in the MD runs, MICAL_MO-CH_ option 1 (yellow cartoon) docked in the electron microscopy model of the F-actin filament (PDB-ID: 3LUE). The CH domain ABS is coloured in cyan. The actin filament is represented as solvent accessible surface with each actin monomer coloured differently. The position of the CH type 2 of the electron microscopy structure of actinin is indicated by a red outline. (**b)** Same drawing from panel **a** with the solvent accessible surface of the CH type 2 domain of actinin coloured dark green. (**c)** Final structure of the actin monomer with the FAD of MICAL_MO-CH_ in the “out” position (oxidized) and (**d)** with the FAD in the “in” position (reduced). See also Supplementary Figure S7 in supporting information.

As no crystal structure of MICAL in complex with F-actin is available, we built a possible model of the complex (Fig. 9a,b), using the MICAL_MO-CH_ crystal structure and a dimer of actin from the most recently reported F-actin model (PDB 2ZWH^23^). The CH domain of MICAL was essential in choosing the initial position and the orientation of MICAL_MO-CH_ on the F-actin; we hypothesize that the CH domain is necessary for optimizing the binding of the MO to oxidize Met 44. The initial position was chosen based on aligning the CH domain of MICAL_MO-CH_ and the CH of actinin bound to actin (PDB 3LUE^24^), followed by manual adjustment to bring both the MO and CH domains in close proximity to the actin dimer (Fig. 9a,b). Although Met 44 is far from the surface, the wide opening of the MICAL’s active site can be oriented towards the D-loop. This model served as a starting point for a series of molecular dynamics simulations in which, in each successive run, actin’s Met 44 was harmonically constrained with a soft force constant (0.5 kcal mol^−1^ Å^−2^) to be closer to FAD-C4a, until its sulphur atom was within ~3 Å of the FAD-C4a.

The simulations reveal that it is possible to attain a D-loop conformation in which Met 44 is in proximity to FAD-C4a (hereafter referred to as “D-loop out” conformation); shown in Fig. 9c,d. A conformation with Met 44 close to the FAD-C4a is possible regardless of whether the FAD of the MO domain is in the oxidized “out” or the reduced “in” conformation. The potential energy, averaged over the production phase of the simulation, of the “D-loop out” conformation is within 3% of that of the original conformation^23^ (Table 5). The major structural change in the actin monomer is in the D-loop itself and the few residues surrounding it; the rest of the actin monomer remains close to the original structure (Fig. 9c,d). The fact that this “D-loop out” conformation can be reached in a molecular dynamics simulation, using gentle steering and without the need to break bonds or significantly alter the integrity of either structure, indicates that this conformation can be populated with a high enough frequency to be kinetically competent.

## Discussion

Our studies provide novel insights into the role of the MICAL_CH_ domain in modulating the catalytic activity of MICAL towards F-actin. This work also reports the first example to date of a CH domain modulating the activity of an adjacent *catalytic* domain.

In the two crystal structures of the MICAL_MO-CH_ presented here, the structures of the MO domain and the CH domain are similar to those of the isolated domains. As no electron density is found for the linker connecting the two domains, which of the CH domains in the asymmetric unit is connected to the MO domain cannot be decided from the structure.

To resolve the ambiguity, SAXS data of MICAL_MO-CH_ in solution were collected. *The ab-initio* low-resolution envelopes and rigid-body refinement using the SAXS scattering data (Fig. 7) indicate that in solution MICAL_MO-CH_ is an elongated molecule that resembles crystallographic option 1 (Fig. 6a) but with a slightly larger separation between domains (Fig. 7d). This larger separation is compatible with a flexible linker between the two domains. The fact that none of the possible MO-CH arrangements are identical between the two crystal forms also supports the argument that there is conformational flexibility between the two domains.

What is immediately evident upon inspection of the structures is that the CH domain does not significantly alter the conformation of the MO domain nor does it obstruct the active site, regardless of the choice of asymmetric unit (Fig. 1c).

A characteristic feature of this monooxygenase family is that binding of the oxygen-acceptor substrates (i.e. F-actin) accelerates NADPH oxidation. In these enzymes the loss of reducing equivalents by production of peroxide is actively suppressed; however, leakage velocities between 1 and 2 s^−1^ are frequently observed. Both MO and MO-CH show low turnover rates (0.7 and 1.7 s^−1^, respectively) in the absence of F-actin. The suppression is relieved by the oxygen acceptor substrate and accelerations between 10^2^–10^5^ fold have been observed in aromatic monooxygenases^25^.

Acceleration of NADPH oxidase activity in the presence of similar amounts of F-actin for different MICAL domain combinations and species have been reported: acceleration of 5-fold for *human* MICAL_MO_ in presence of 2.4 μM of F-actin^26^, 35-fold i in *Drosophila* MICAL_MO-CH_ in presence of 2.3 μM *Drosophila* F-actin^17^, and recently a 10-fold across all human MICAL_MO-CH_ types (MICAL-1, 2, and 3) in presence of 2.8 μM F-actin^27^. In contrast, we observed moderate 1.7-fold increase for our mouse MICAL_MO_ and 4.7-fold for the MICAL_MO-CH_ construct in the presence of 2 μM of F-actin (Table 4). Different degrees of inhibition by the buffer used in the experiments —we choose to maximize F-actin polymeric state— or differences between species may be the cause of the discrepancies observed. There is agreement nevertheless, between our data, the human data, and the *Drosophila* MICAL_MO-CH_ in that all three show acceleration in the rate of NADPH oxidation in the presence of F-actin.

Interestingly, the reducing equivalents consumed in the time interval used for velocity determination (5–10 s) exceed the amount of activator present in some conditions (for example for 0.4 μM of F-actin; Table 4). The NADPH oxidation profiles (Supplementary Fig. S4) show an early deviation (curvature) from the initial slope that may be associated with a second phase with depleted activator. The NADPH oxidation rate by *Drosophila* MICAL is 0.8 μM of NADPH per second (estimated from Fig. 1F of ref. 17 using the described sampling of 10 s). There dMICAL would have processed sufficient reducing equivalents at the first data point to oxygenate all the F-actin (2 μM) present in the reaction several times, suggesting that either (a) not all the NADPH consumed is used to modify the bound F-actin, (b) there is more than a single site of modification per F-actin molecule, or (c) the modified F-actin continues to activate the redox reaction independent of or in addition to being a substrate.

Overall, MICAL_MO_ and MICAL_MO-CH_ catalyse the oxidation of NADPH faster (β > 0) in presence of F-actin but with different kinetic characteristics (Fig. 8). There are significant differences between catalytic activities (*k*_cat_^MO^ < *k*_cat_^MO-CH^) and acceleration factors (β^MO^ < β^MO-CH^), consistent with further enhancement of the redox activity with F-actin when the CH domain is present. In the range of F-actin concentrations tested, the apparent redox catalytic power increases more than 20-fold in the case of MICAL_MO-CH_; in contrast to the modest 3-fold increase observed in the case of MICAL_MO_ (Table 4). F-actin accelerates NADPH oxidation more for MICAL_MO-CH_ than for MICAL_MO_ (β = 7.4 ± 1.3 *vs*. 4.9 ± 0.4; Fig. 8c). The CH domain does not change the affinity for each substrate alone —*K*_M_^MO^ is not significantly different from *K*_M_^MO-CH^ and *K*_A_^MO^ ≈ *K*_*A*_^MO-CH^; Fig. 8d. The difference between the two proteins, MICAL_MO_ and MICAL_MO-CH_, is found in the kinetics of oxidation of actin. In the case of the single MO domain, F-actin functions as a simple non-essential activator that provides an alternative path for the oxygenation reaction to occur, resulting in an increase of the NADPH oxidation rate without changes in NADPH affinity (α = 1). In contrast, in MICAL_MO-CH_, in addition to this effect, F-actin modifies the MO active-site in a way that increases the affinity for NADPH (α = 0.16) and its oxidation rate. These observations suggest that the CH domain functions by coupling second substrate-binding to enzymatic rate enhancement.

Of the possible MO-CH arrangements in the crystal asymmetric unit, option 3 shows the most compact structure with the closest and the tightest interactions between the MO and CH domains (Fig. 6c). The characteristics of this interaction—buried area (≈1000 Å^2^), charge complementarity, and the degree of conservation of the interface residues among species and isoforms—suggest that this contact may have a physiological role as an intermolecular interaction between the MO and the CH domains. MICAL-2 and MICAL-3 have longer linkers and high residue conservation in the putative MO-CH interface (Fig. 5c), providing additional indirect evidence that these interfaces may be a hot spot for a protein-protein association with biological relevance in these isoforms.

The failure of option 3 to explain the SAXS results (Table 3, Fig. 7) argues against this being the actual arrangement between the two domains in solution. However, the CH domain placement far away from the active site in all the other options makes it physically improbable that the covalently connected CH domain may have a direct effect on the MO activity.

This apparent contradiction may be explained by a *trans* cooperative mechanism in which the CH domain from one MICAL_MO-CH_ molecule interacts with the MO domain of another MICAL via the option 3 interaction. This interaction is particularly suited for *trans* binding because the *cis* interaction is unfavorable given the length of the linker peptide (see above). Actin depolymerization factors ADF/Cofilins are a well-characterized family of filament-severing proteins that also show a common cooperative mechanism of action^28^.

In summary, the structures in crystal and solution of MICAL_MO-CH_ presented in this study provide structural insight into the function of MICAL’s CH domain. The CH domain couples binding of F-actin to catalytic site modification enhancing the monooxygenase activity. In this CH-mediated mechanism, cooperative binding of MICAL molecules, perhaps using the contact observed in option 3, appears to be involved. This cooperative binding further stabilizes the formation of the MICAL/F-actin complex, while at the same time allowing the non-covalent concatenation of MICAL molecules on the surface of the filaments that will significantly increase the efficiency of actin modification by MICAL. The contact between the CH domain and the catalytic site loop Lc (Fig. 5b) provides a mechanism for modulating both substrate affinity and catalytic activity.

Our F-actin/MICAL_MO-CH_ model suggests that direct oxidation of actin Met 44 by MICAL is possible; however, it does not rule out that oxidation of Met 44 occurs by a high local H_2_O_2_ concentration in the cavity formed upon binding MICAL_MO-CH_ to actin. Restricted access of the H_2_O_2_ to the sulfur atom of Met 44 may result in an apparent stereospecificity^29^,^30^. The model for oxidation of F-actin by MICAL presented here involving a large change in the conformation of the D-loop of actin will be important for guiding future experiments aimed at elucidating mechanistic details of the redox reaction catalyzed by MICAL.

## Methods

### Cloning

A plasmid containing DNA coding for residues 2–615 of MICAL-1 from *Mus musculus*, codon-optimized for expression in *Escherichia coli was obtained from* Genescript Inc. In addition of a Gly from the expression vector digestion site, the Q78K substitution was introduced to remove an endogenous protease site. This construct, cloned onto a pET28a expression vector containing an N-terminal His-tag with an engineered N-terminal *Tobacco Etch* virus (TEV) protease site, was used to transform *Escherichia coli* BL21.

### Protein expression and purification

After induction of the transformed *E. coli* cells by addition of 0.2 mM isopropyl-β-D-thiogalactoside (IPTG), cells were grown for 15 hours at 17 °C in LB media before harvesting by centrifugation. Cells were resuspended in lysis buffer (50 mM Tris-HCl pH 7, 140 mM NaCl, 10 mM imidazole, 0.1% Tween-20, 5 mM MgCl_2_, 2 mM β-mercaptoethanol, 5 mM benzamidine, and 10% v/v glycerol) and broken with a microfluidizer. After centrifugation and filtering, the protein was purified by Ni-Sepharose affinity chromatography with a gradient of 10–500 mM imidazole in 50 mM Tris-HCl pH 7.3, 140 mM NaCl. The eluted protein was dialyzed against 50 mM Tris-HCl pH 7.0, and 200 mM Glycine, and digested overnight with TEV protease to remove the His-tag. The cleaved product was purified using a Source 15S cation exchange column (GE Healthcare), eluting with 50 mM Tris-HCl pH 7.0, and a 0–1 M NaCl gradient. The final yield was ~3 mg per liter of culture. MICAL_MO-CH_ was concentrated to ~25 mg/ml using a 3 kDa MWCO ultrafiltration device (GE Healthcare), before storage at −80 °C.

The mouse MICAL_MO_ (1–484) used in the enzyme kinetic experiments described below was prepared as previously described^12^.

### Crystallization (Native 1)

1 μl of MICAL_MO-CH_ protein solution (25 mg/ml in 50 mM Tris-HCl pH 7.0, 200 mM NaCl, 2 mM DTT, 2% v/v glycerol) was mixed with 1 μl of a 19.0 mM peptide solution of actin’s D-loop (39-RHQGVMVGMGQKDS-52) and incubated at room temperature for 30 min. Two μl of this solution, combined with 1 μL of reservoir solution (100 mM HEPES pH 7.0, 20% PEG 2000 MME) and equilibrated against 500 μl of reservoir solution, produces small crystals of poor diffracting quality. Single crystals suitable for data collection were obtained by a two additional steps: 1) drops were micro-seeded by hair-streaking of the crystallization drops after a day of equilibration after touching the previous crystals with the hair; and 2) a new crystallization round mixing protein aliquots with a seed dilution generated by smashing a few of the previously obtained crystals in reservoir solution. After this final step, suitable single crystals grew in 24 h at 20 °C.

### Crystallization (Native 2)

For crystallization, the protein solution (25 mg/ml) was prepared in a buffer containing 100 mM sodium citrate pH 5.0, 200 mM NaCl. Differential scanning fluorimetry showed high stability of the protein in this buffer condition (data not shown). 1 μl of this protein solution was combined with 1 μl of microseeds diluted in reservoir solution [100 mM HEPES pH 7.0, 20% PEG 2000 MME] and equilibrated against 500 μl of reservoir solution. Crystals grew in 24 hours at 20 °C.

### Crystal Data Collection, Structure Determination, and Refinement

Cell parameter variations among crystals obtained in the same conditions during the search for diffraction quality crystals were frequently observed (data not shown). Two crystal forms showing differences in the ***c*** cell-dimension and β angle were selected for analysis. Diffraction data from both crystal forms (native 1 and native 2) frozen in the their mother liquors, with 20% v/v glycerol added as cryoprotectant, were collected using a Saturn 944 + CCD as a detector. The source was an FR-E + Super-Bright^*TM*^ copper rotating anode x-ray generator equipped with VariMAX™ mirrors for monocromatization and collimation (Rigaku Americas Corporation, The Woodlands, TX). Data were processed with HKL2000 (HKL Research Inc.). The structure of native 1 was determined by molecular replacement with the program MOLREP as implemented in the CCP4 Suite^31^, using the previously determined structure of MICAL_MO_ (residues 1–484) as a search model (PDB 2BRA^12^). The model of the CH domain was built manually with the program COOT^32^ on the unassigned electron density of a sigmaA-weighted difference Fourier map (mF_O_-DF_C_) calculated with phases from the MO domain alone. The final structure was refined using the program REFMAC in CCP4 Suite). The structure of the second crystal was determined by molecular replacement using the two domains of the first crystal as separate search models. The models were refined by rigid body refinement, followed by restrained refinement and TLS/Restrained refinement (Fig. 1). All data collection and refinement statistics are presented in Table 1.

### Size exclusion chromatography

The oligomeric size of MICAL_MO-CH_ in solution was determined using size exclusion chromatography (SEC) with a Superose™ 12 10/300 GL column (GE LifeSciences) in a buffer containing 50 mM Tris-HCl pH 7.0 and 500 mM NaCl. Five molecular standards of known size (Thyroglobulin, Globulin, ovalbumin, myoglobin, and vitamin B12) were used for calibration in an independent run. The elution position of the MICAL_MO-CH_ peak compared to those of the five standards (Fig. 2a) was used to estimate the molecular weight (Mw) using the relation between the log(Mw) and the ratio between the sample elution volume (Ve) and the column void volume (Vo) (Fig. 2B).

### Small angle X-ray scattering (SAXS) of MICAL_MO-CH_

SAXS data were collected at the SIBYLS beam line (B12.3.1) of the ALS for q values in the range 0.0128–0.3253 Å^−1^ using three protein concentrations (2, 4 and 7 mg/ml) in a buffer containing 50 mM Tris HCl pH 7.0, 200 mM NaCl, 2 mM DTT, and 2% v/v glycerol (Table 3). The particle molecular mass was estimated by three independent methods (Table 3 and Suppl. Fig. S4): a) using the relation between the intensities of the scattering at zero angle [I(0)] of the MICAL_MO-CH_ sample and that of a standard -dimeric bovine serum albumin (BSA; Mw: 132 kDa, SIGMA Inc.)- at the same three concentrations used for the protein sample; b) using the saxsmow web server estimator (http://www.ifsc.usp.br/~saxs/saxsmow.html)^33^; and c) using the relation between the molecular weight and the ratio of the square of the correlation volume (Vc) to R_g_ as implemented in Scatter 2.0^34^. An *ab-initio* low-resolution model of MICAL_MO-CH_ was obtained by a two-step procedure. In the first step, twenty envelopes were generated by DAMMIN (ATSAS) and averaged with DAMAVER (ATSAS) to provide a low-resolution dummy atom model (DAM) with an overall normalized spatial discrepancy (NSD) of 0.7^35^. In a second step, the averaged DAM generated by DAMAVER was used as a starting model for a final run of DAMMIN. The final low-resolution model (Fig. 7) has an averaged NSD of 0.47 with a standard deviation of 0.08. In addition, an all atom-model, obtained by rigid-body refinement of the two separated MICAL_MO-CH_ domains against the experimental scattering using the program SASREF^36^, reached a χ^2^ of ≈0.69 (Fig. 7).

### Rate of NADPH oxidation as a function of F-actin concentration

MICAL’s constructs redox activity in presence of F-actin was measured as NADPH oxidation monitoring the decrease in absorbance at 340 nm over time (Fig. 8, Supplementary Fig. S5). The reaction was initiated with the addition of the enzyme to a 100 μL quartz-cuvette and mixing for 5 sec by slowly pipetting to avoid bubbles. To maintain steady-state conditions in NADPH and F-actin, the rate of reaction was measured as close as possible to the beginning of the reaction, commonly after 5 sec and before 10 sec. The initial slope of the curve was taken as the initial reaction-rate of NADPH oxidation (***v***) (See Supplementary Fig. S5 for a example of the observed curves). Kinetic data for MICAL_MO_ and MICAL_MO-CH_ were obtained at different NADPH concentrations (six data points ranging from 3 to 100 μM for the MO and five data points from 10 to 150 μM for the MO-CH) for each F-actin concentration used (five conditions 0.0, 0.4, 0.9, 2.2, and 7.5 μM for the MO and four conditions 0.0, 2.0, 4.0, and 8.0 μM for the MO-CH). Each data point was repeated three to four times and the mean ***v*** and its standard deviation recorded. Data from each construct were fitted using the general steady-state kinetic relation in the presence of a non-essential activator known as a Henri-Michaelis-Menten relation given by the equation (1).
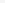





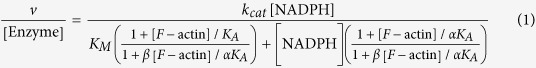




in which *K*_*M*_ and *K*_*A*_ are the equilibrium constants in the quasi-equilibrium conditions depicted in Fig. 8a for NADPH (substrate) and F-actin (activator), assuming non-limiting amounts of these, α is the ratio between substrate affinities of the free enzyme and activator-bound enzyme, β is the acceleration factor produced by the activator (F-Actin), and 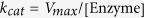 the turnover number, where 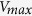 is the initial reaction rate at infinite concentration of substrate. Bracketed variables are the concentration of the reaction components. Evaluating equation (1) at a given concentration of F-actin simplifies to the relation for a simple enzyme-substrate reaction




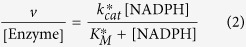




In which the apparent NADPH dissociation constant (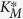) and catalytic rate (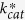)—now functions of the activator concentration—are related to the absolute constant parameters in equation (1) by:




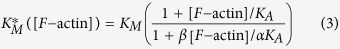







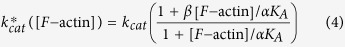




The turnover numbers (*k*_cat_) of MICAL_MO_ and MICAL_MO-CH_ at different concentrations of F-actin are shown in Fig. 8. Kinetic parameters were determined by non-linear least-squares fit of 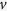, to the equation (1) using the program Prism6 (GraphPad Inc.), which was also used for the statistical analysis. Human non-muscle G-actin (from human platelets; #APHL99, Cytoskeleton Inc.) was polymerized following manufacturer protocols. The polymerization buffer: 5 mM TrisHCl pH 8.0, 50 mM KCl, 2 mM MgCl_2_, 0.2 mM CaCl_2_, 0.5 mM DTT, 1 mM ATP was used in the steady-state kinetic experiments.

The concentration of the MICAL constructs used (600 nM) was determined by absorbance at 280 nm of a sample denatured in 6 M GdnHCl using the Gill and von Hippel method^37^.

The statistical analysis were performed using an F-square sum F-test for each dataset fit with Equation (2) as is implemented in the program Prizm® v 6.0.

### Modelling of the MICAL_MO-CH_ complex with actin

A model of the complex of MICAL_MO-CH_ and F-actin was made using the crystal structure of MICAL_MO-CH_ reported in this study and a dimer of actin from the most recent F-actin model (PDB 2ZWH^38^). In the case of the reduced enzyme, the MO in the MICAL_MO-CH_ structure was replaced by the one in the crystal structure of the MO with the reduced FAD (PDB 2C4C^13^). To obtain a starting model, the CH domain of MICAL_MO-CH_ was aligned to the CH domain of an electron microscopy model of actinin bound to F-actin (PDB 3LUE^24^), followed by manual adjustment of MICAL_MO-CH_ to orient the large opening of the active site in the MO towards the D-loop (DnaseI-binding loop) of actin which contains Met 44, while maintaining the contact between the CH domain and actin. In these conditions, the closest distance between Met 44 and the C4a of the FAD is ~32 Å. For direct oxidation to be possible, the D-loop must adopt a conformation in which the sulfur atom of Met 44 is within ~3 Å of the distal oxygen of the C4a-hydroperoxyflavin FAD intermediate (FAD-C4a-O-OH) of MICAL’s active site. To assess whether such a conformation is possible, we used a series of steered molecular dynamics (MD) calculations. CHARMM version 28b2 with the CHARMM 28b2 force field was used in the computations with implicit solvent and a distance dependent dielectric constant. The models were optimized by minimizing the energy for 1000 cycles of steepest descent, followed by 1000 cycles of conjugate gradient, and finally 1000 cycles of adopted-basis Newton-Raphson minimization. For all MD calculations, the FAD cofactor was harmonically constrained to either the oxidized “out” conformation or the reduced “in” conformation with a soft force constant (1 kcal mol^−1^ Å^−2^). Leapfrog Verlet molecular dynamics simulations were performed at a constant temperature of 300 K and ran for 30,000 fs, harmonically constraining Met 44 to a chosen position with a force constant of 0.5 kcal mol^−1^ Å^−2^). In each successive simulation, Met 44 was constrained to a position closer to the FAD-C4a atom, until the D-loop achieved a conformation where Met 44 was within the 3 Å range required for oxidation to be possible. The average value of the energy and its fluctuations during the last 10,000 fs were calculated with an in-house written program.

## Additional Information

**How to cite this article**: Alqassim, S. S. *et al*. Modulation of MICAL Monooxygenase Activity by its Calponin Homology Domain: Structural and Mechanistic Insights. *Sci. Rep*. **6**, 22176; doi: 10.1038/srep22176 (2016).

## Supplementary Material

Supplementary Information

## Figures and Tables

**Table 1:** MICAL_MO-CH_ data collection and refinement statistics.

Crystal	Native 1	Native 2
Wavelength (Å)	1.5416	
Resolution range (Å)	32.14–2.309 (2.392–2.309)	26.81–2.878 (2.981–2.878)
Space group	P 1 2_1_ 1	P 1 2_1_ 1
Unit cell	71.9 49.9 95.85 90 97.0 90	70.2 50.2 97.0 90 101.2 90
Measured reflections	93863(28540)	52018(14602)
Unique reflections	28284 (2316)	14591 (1005)
Multiplicity	3.3(2.0)	3.6(2.7)
Completeness (%)	94.25 (78.27)	95.03 (65.51)
Mean I/sigma	17.0 (1.2)	6.6 (1.1)
Wilson B-factor	38.33	36.27
R-sym	0.08 (0.49)	0.18 (0.64)
R-work	0.22 (0.27)	0.20 (0.35)
R-free	0.28 (0.37)	0.27 (0.44)
N^o^ of non-hydrogen atoms	4776	4608
macromolecules	4518	4510
ligands	61	60
water	197	38
Protein residues	583	581
RMS(bonds)	0.010	0.012
RMS(angles)	1.26	1.35
Ramachandran favored (%)	96	92
Ramachandran outliers (%)	0.69	0.52
Average B-factor	52.70	41.60
macromolecules	53.40	41.90
ligands	38.40	29.00
solvent	41.00	19.40
PDBIds	4TXI	4TTT

^#^The change in the β angle is the largest difference between the two crystal forms.

**Table 2:** Buried surface area between the MO and CH domains in each connectivity option.

Option	Native 1 (Å^2^)	Native 2 (Å^2^)
1	89.8	141.2
2	120.1	261.4
3	1015.0	901.2
4	130.2	138.1

**Table 3:** MICAL_MO-CH_ SAXS Data Collection and Scattering refinement parameters.

**Data collection parameters**			
Instrument	Beam line SIBYL (LNBL ALS B12.3.1)		
q range [Å^−1^]	0.0128 - 0.3253		
Exposure times [sec]	0.5, 1.0, 2.0, 4.0		
Concentration range (mg ml−^1^)	2.0	4.0	7.0
Structural parameters (from P(r))			
I(0) [cm^−1^]	85.4 (80.1)	216 (199)	413 (398)
R_g_ (Å) [from P(r)]	31 (31.5)	31.7 (32.4)	32.5(34.1)
D_max_ [Å]	114	120	120
Porod’s volume estimate [x 10^3^ Å^3^]	116(112)	111(103)	107(101)
Dry volume calculated from sequence [Å^3^]	81884		
**Particle-mass estimation/method**			
Molecular Mass M_r_/from I(0); BSA STD [Da] (Δm %)*	55000 (−19.6%)		
Molecular Mass M_r_ /sasmow, q < 0.25^33^ [Da] (Δm %)*	71500 (4.4%)		
Molecular Mass M_r_/Mw = (Vc^2^ Rg^−1^/1.231)^34^	51500	53600	55000
Molecular Mass/Size Exc. Chr. [Da] (Δmass %)*	61455 (−10.2%)		
Calculated M_w_ from sequence + FAD [Da]	68425		
**Final NSD of the *ab-initio* model (σ)**	0.47(0.08)		
	Model R_g_ (Å)	Fit SAXS χ^2^	
Sasref rigid-body solution	30.4	0.98	
Option 1	28.5	1.5	
Option 2	27.5	2.2	
Option 3	24.3	4.2	
Option 4	26.8	2.8	
**Software employed**			
Primary data reduction	At the beam-line		
Data processing	Scatter 2.01c		
*Ab-initio* analysis	DAMMIN^35^		
Validation and averaging	Damaver (ATSAS)		
Rigid-body modeling/refinement	Sasref^36^		
Computation of scattering profiles	FOXS^42^		
Three dimensional graphic representations	Chimera^41^,^44^/PyMol^45^		

See also Supplementary Fig. S4. *Difference with respect to the theoretical mass.

**Table 4:** Apparent 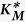, 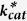, and catalytic power (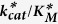) for both protein constructs.

	MICAL_MO_				
[F-actin] μM	0.0	0.45	0.9	2.2	7.5
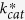 sec^−1^	0.68 ± 0.02	0.71 ± 0.03	0.80 ± 0.04	1.18 ± 0.08	1.72 ± 0.11
μM	28.8 ± 2.2	23.3 ± 2.9	24.0 ± 2.8	26.2 ± 4.5	25.5 ± 3.9
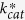 sec^−1^ mM^−1^	23.6	30.5	33.0	45.5	67.4
	**MICAL**_**MO-CH**_				
[F-actin] μM	0.0	2.0	4.0	8.0	
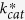 sec^−1^	1.7 ± 0.5	7.9 ± 0.4	10.0 ± 0.2	10.3 ± 0.15	
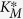 μM	37.7 ± 8.4	23.9 ± 5.1	17.5 ± 3.0	9.9 ± 2.0	
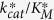 sec^−1^mM^−1^	45.1	330.5	571.4	1040.4	

**Table 5:** Average energies from molecular dynamics simulations of MICAL_MO-CH_ and F-actin.

	Initial	D-loop out (FAD oxidized)	D-loop out (FAD reduced)
Bond	1004.31	1008.50	1006.45
Angle	2560.50	2563.94	2556.54
Dihedral	1930.89	1917.22	1905.48
Improper	177.15	180.15	179.40
van der Waals	−1804.69	−1869.97	−1856.41
Electrostatic	−593.64	−628.71	−616.30
Harmonic	3.97	4.85	3.47

All energies reported are in kilocalories per mole.
