# Supplementary material for: Modulation of MICAL Monooxygenase Activity by its Calponin Homology Domain: Structural and Mechanistic Insights
Source: Sci Rep. 2016 Mar 3;6:22176. doi: 10.1038/srep22176 (PMC4792234; doi:10.1038/srep22176)

Supplementary Material

Modulation of MICAL Monooxygenase Activity by its Calponin Homology Domain: Structural and Mechanistic Insights

Saif S. Alqassim[1], Mauricio Urquiza, Eitan Borgnia, Marc Nagid, L. Mario Amzel[1*], and Mario A. Bianchet[1,2*]

Supplementary Figures Captions

Supplementary Figure S1 | Structural alignment between MO domains. Overlap of the MO domain (PDBid 2BRA) in magenta with that of $MICAL_{MO\text{-}CH}$ reported here in yellow**.** The MO domains overlap with an RMSD of 0.5 Å for 422 Cα atoms.

Supplementary Figure S2 | Sequence alignment of mouse, human, zebra fish, and fly MICAL-1 sequences. Residues in red boxes are completely conserved among species. The residues participating in the interaction between the MO and CH domains of the option 3 are marked with triangles, colored red and blue in the MO and CH domain, respectively. The Actin binding sequence (ABS) and PIP binding region (PBS) are colored cyan and green, respectively, only in the mouse sequence. The missing linker is indicated by a yellow bar below the sequence. The secondary structure elements observed in the structure reported here are displayed above the sequences.

Supplementary Figure S3 | Small Angle X-ray Scattering Data Analysis. (a) Molecular weight estimation of $MICAL_{MO\text{-}CH}$ from the scattering intensity, I(0), vs concentration. Dimeric BSA (*Sigma*-Aldrich™ A8654; Mw 132 kDa) was used as standard at the equal concentrations of $MICAL_{MO\text{-}CH}$ (2, 4, and 7 mg/mL). **(b)** Overlap of the scaled experimental intensity profiles in function of q^{-1} (nm^{-1}) for the three different concentrations used. Neither agregation, radiation damage, nor concentration dependence effects are observed. **(c)** D_{max} determination from the pairwise distance distribution (P(r)) for the three concentrations showing a $D_{max} \approx 120$ Å. **(d)** Scaterng intensity profile fitted by the calculated P(r)s (solid red lines). The three I(q) curves correspond to the 2 mg/mL (green), 4 mg/mL (blue), and 7 mg/mL (cyan) samples with 4 sec exposures. **(e)** Kratky's Plot (q^2 I(q) vs q^2) is compatible with a folded protein (plateu at high q) with some flexibility. **(f)** Agreement between the different structural choices calculated (solid lines) and the observed I(q) (scattered dots). **(g)** Low q region of I(q). **(h)** Agreement between the calculated DAMMIN *ab-initio* envelope and the averaged experimental intensity profile.

Supplementary Figure S4| Steady state kinetics profiles. (a) $MICAL_{MO}$ and **(b)** $MICAL_{MO\text{-}CH}$ time course of the reaction at various concentrations of F-actin. The reaction was followed by monitoring the NADPH UV absorbance at λ 340 nm. 100 nM of enzyme and 100 μM NADPH (*Sigma*-Aldrich™ N8035) were made to react with different amounts of polymerized actin. In the figure the vertical axis is in arbitrary units; for a given [F-actin] each curve was shifted to the same arbitrary initial value of absorbance for better visualization. The rates at 5 sec of the reactions containing $MICAL_{MO}$ **(c)** or $MICAL_{MO\text{-}CH}$ **(d)** are shown by dots; the solid line indicates the average rates. ANOVA was used to estimate significance of the rate increases with respect to the rate in the absence of F-actin.

Supplementary Figure S5| Actin monomer structure before and after the simulation. (a) Before, **(b)** after docking the D-loop to the large entrance of the catalytic site, and **(c)** the same to the small entrance.

Supplementary Figure S6 | $MICAL_{MOCH}$ docking to F-actin simulation. (a) Actin dimer (colored blue and green) docked to the large catalytic site entrance. **(b)** enzyme catalytic site showing the FAD (carbon atoms colored in cyan) and the final position of actin's Met 44 (carbon atoms colored green).

Supplementary Figure S7 | Cross-eye Stereo view of the refined electronic density. The 2DFc-mFo sigmaA-weighted map contoured at 1 sigma level (in blue) is shown around the FAD molecule at the active site of the enzyme. The final model is shown in a stick representation with carbon atoms colored in yellow, nitrogen colored blue, and oxygen atoms colored red.

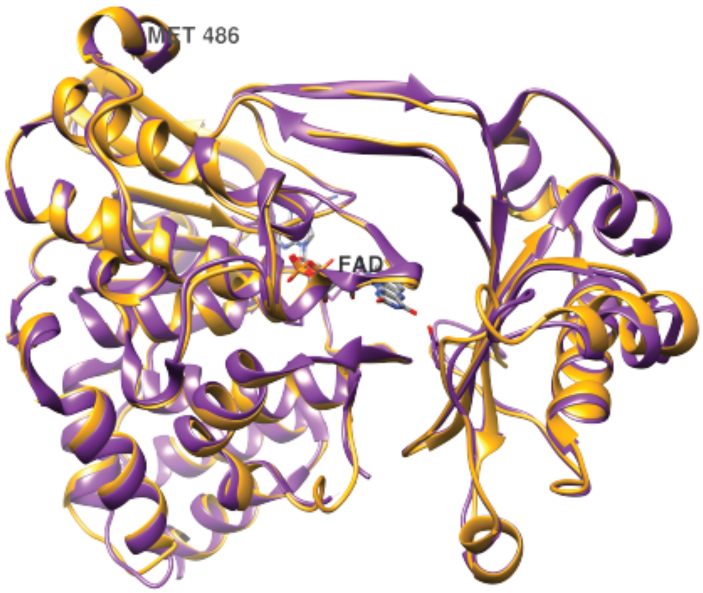

Supplementary Figure S1

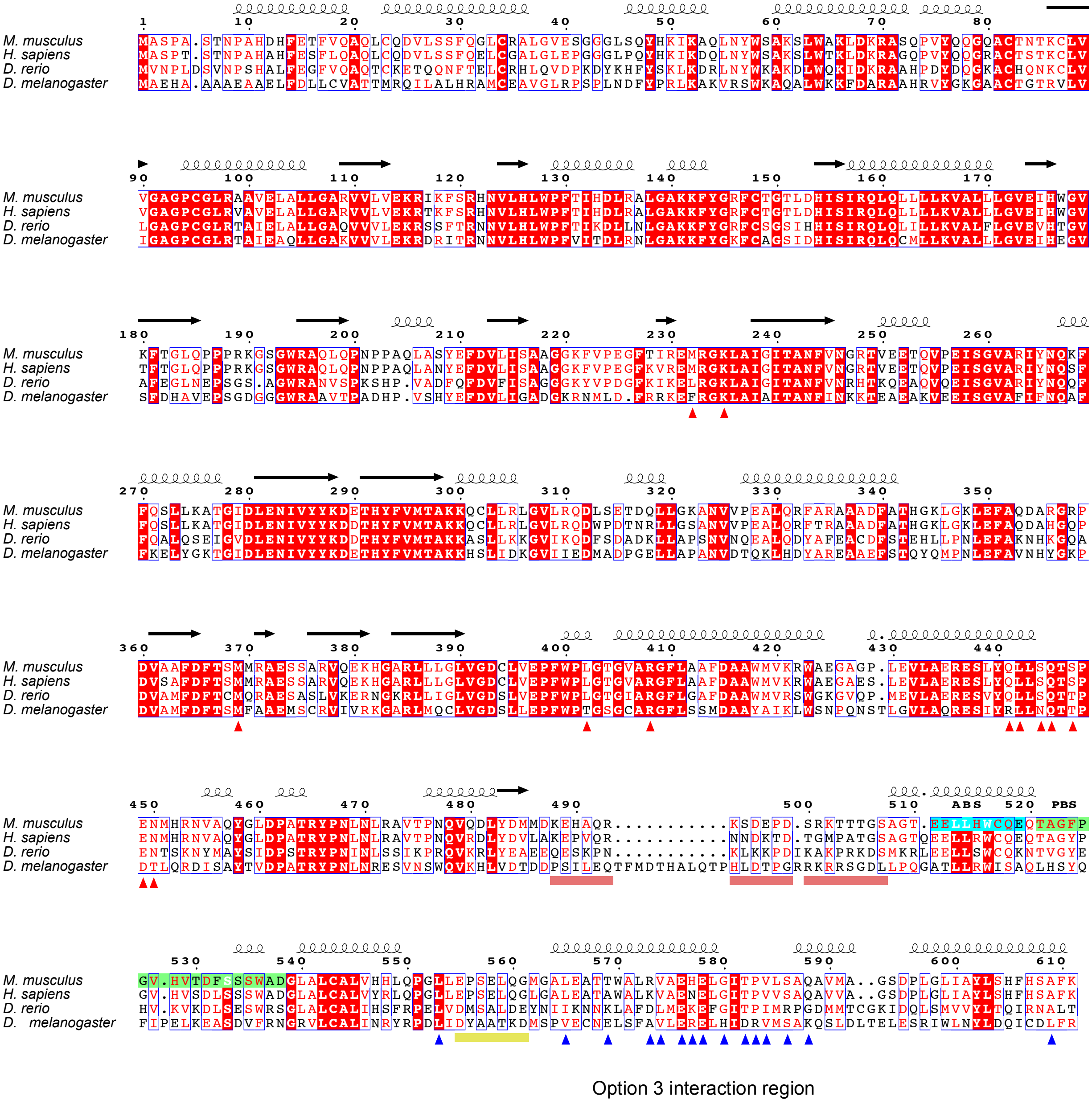

Supplementary Figure S2

Supplementary Figure S3

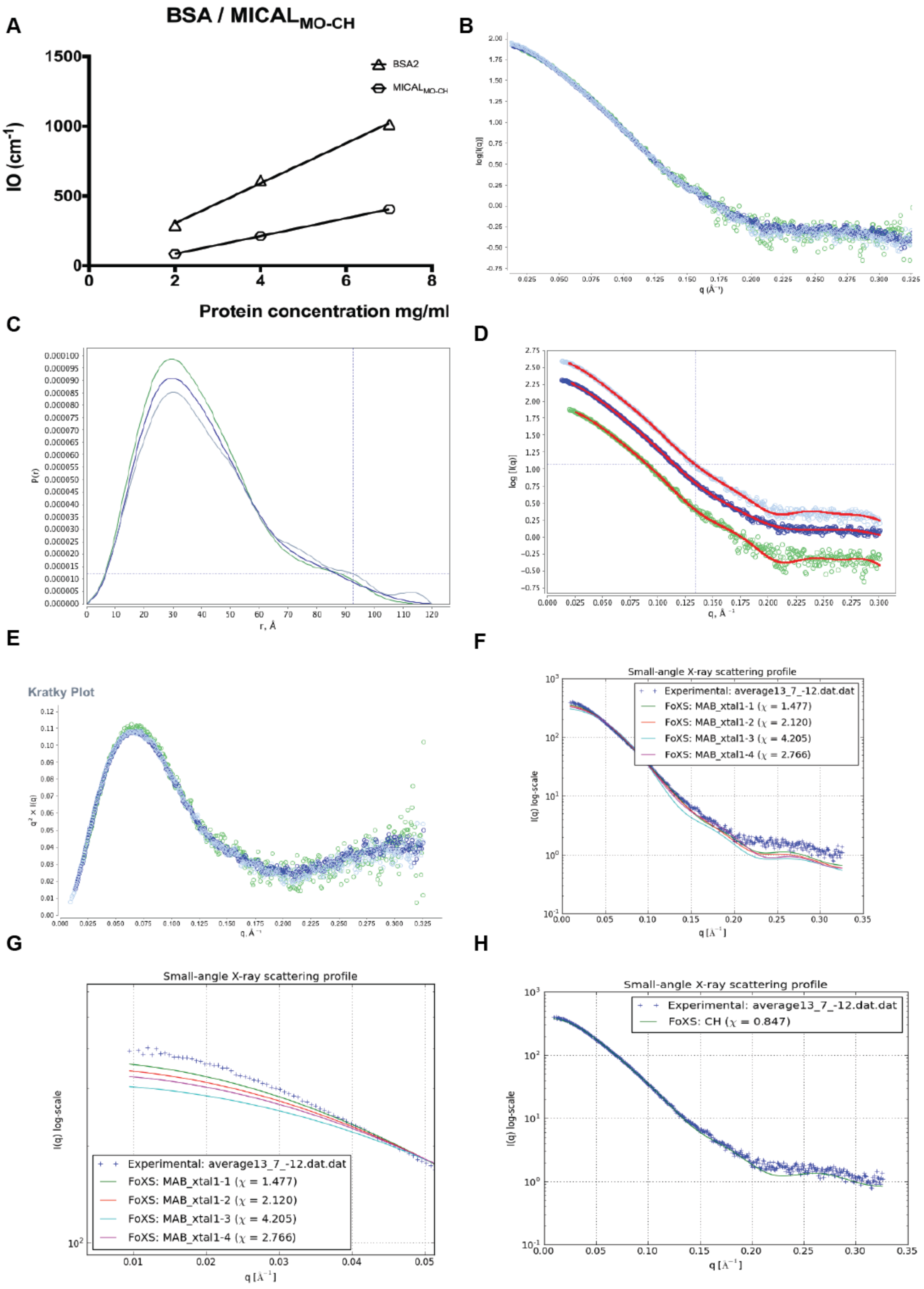

Figure Supplementary S4

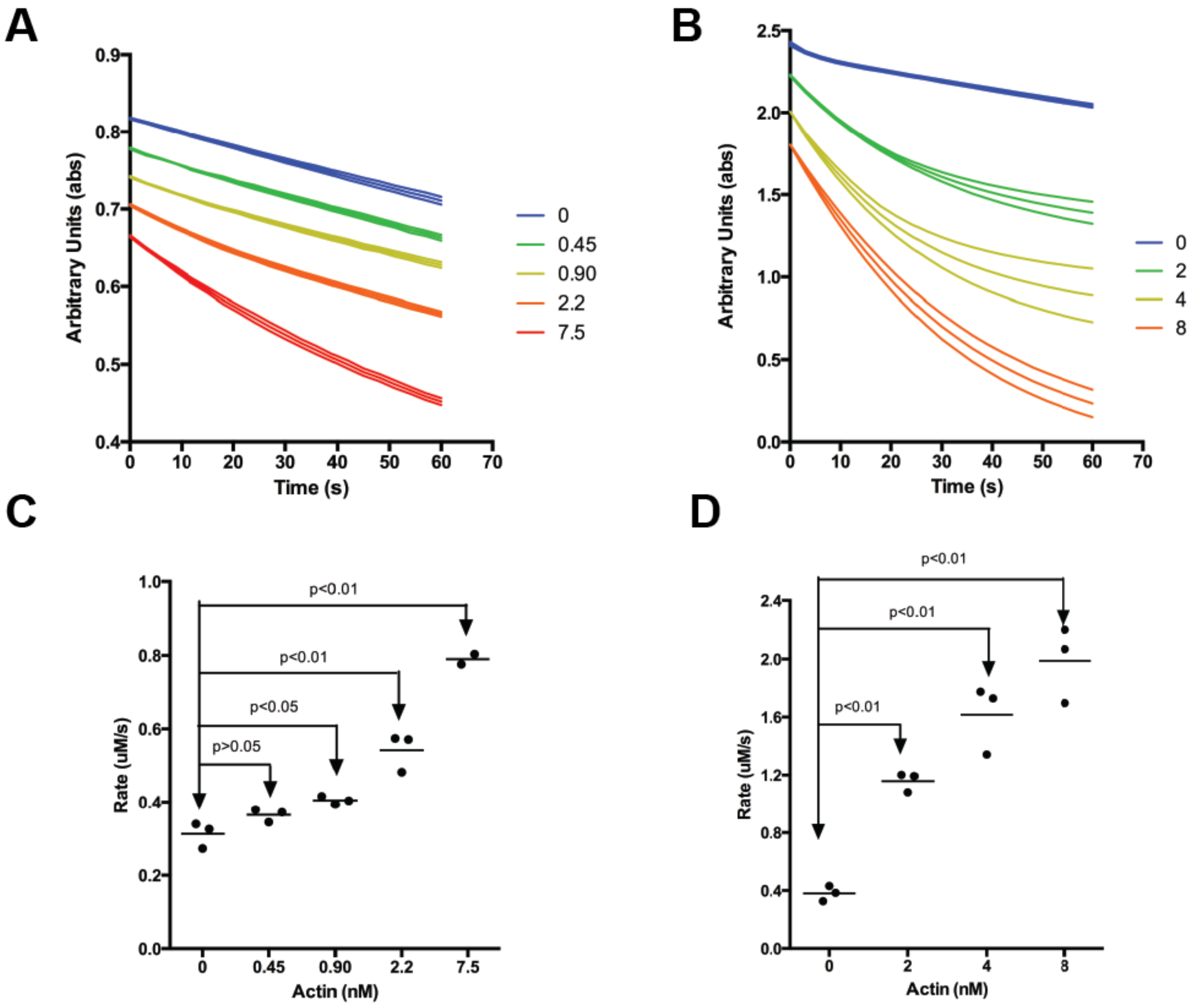

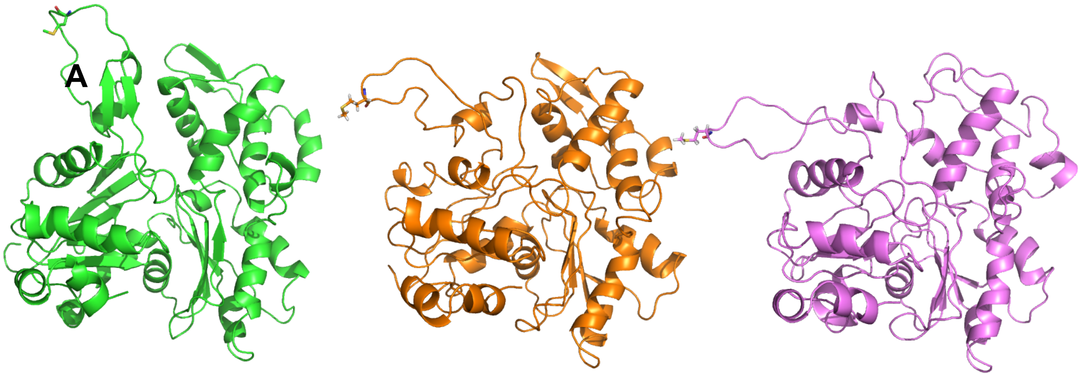

Supplementary Figure S5.

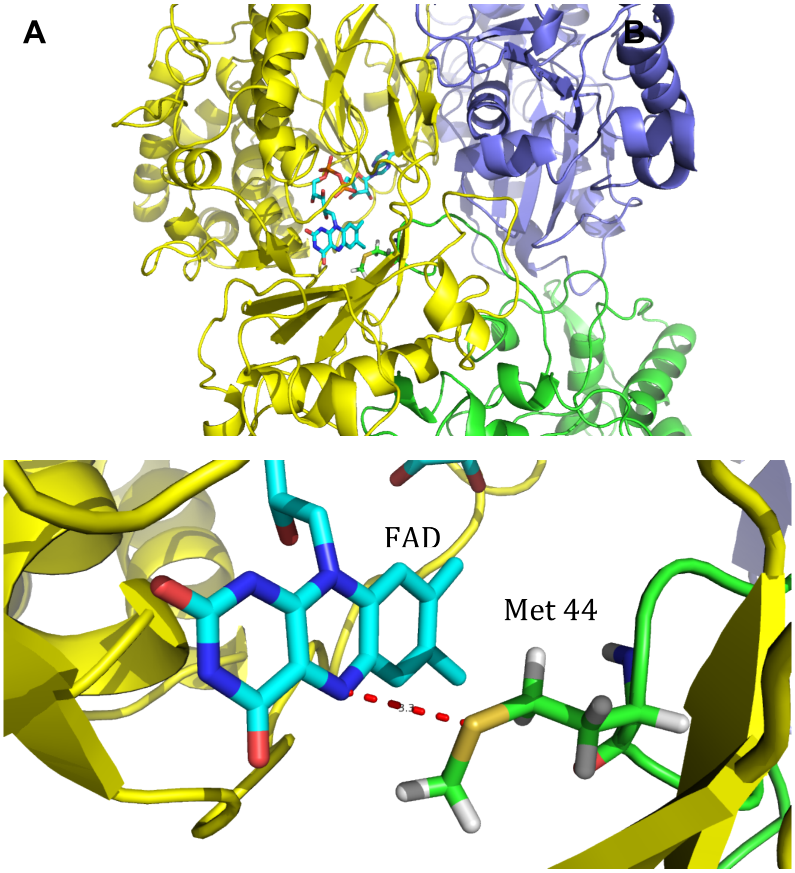

Supplementary Figure S6

Supplementary Figure S7

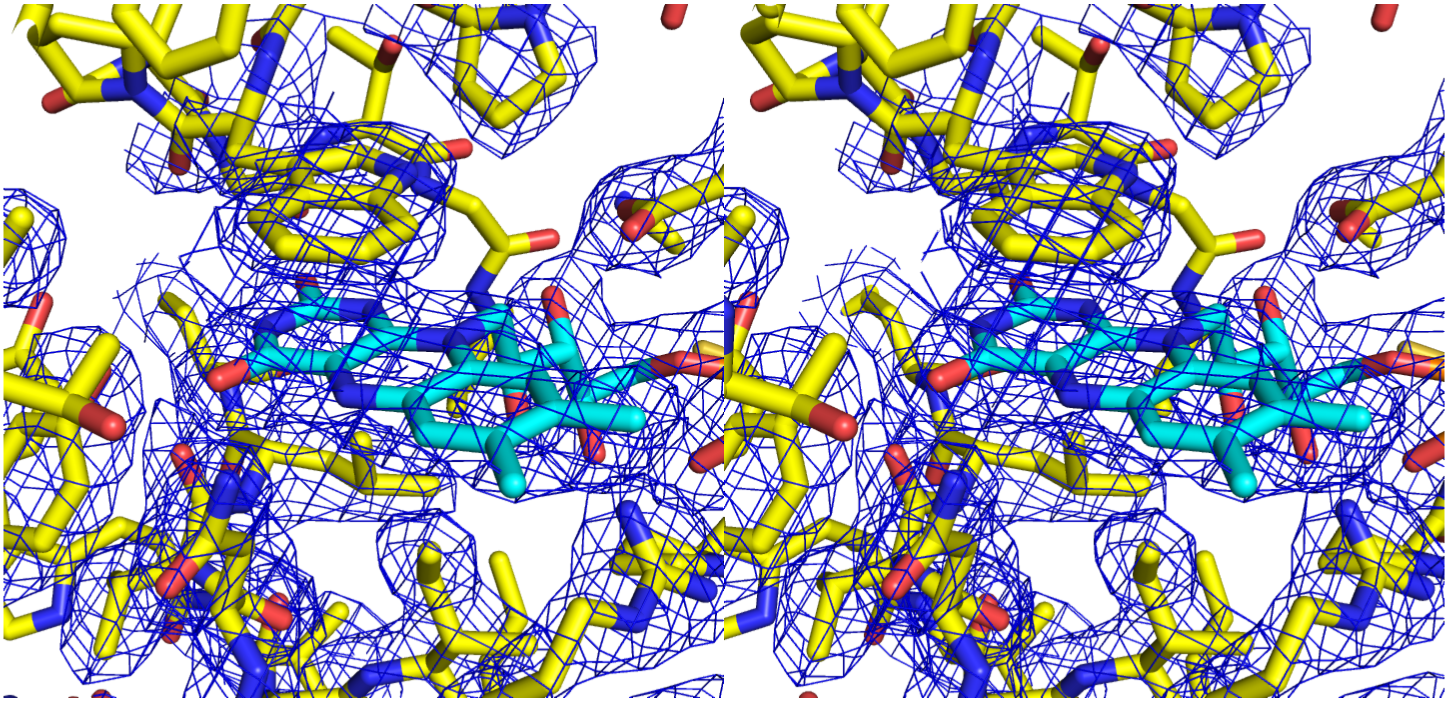

Supplement: Supplementary Information [file srep22176-s1.pdf]
